# Plaque, Prodromes, and Personalized Care: A Case Report Reframing Left Main Coronary Artery Occlusion in Females

**DOI:** 10.7759/cureus.93840

**Published:** 2025-10-04

**Authors:** Colton Herrell, Osadiame C Uduehi, Peter N Rodenko, Alexis C Jablonski, Shashawna S Drum Christie, Josh Elefteratos, Khachig K Ishkhan

**Affiliations:** 1 Medical School, St. George's University School of Medicine, St. George, GRD; 2 Medical School, Washington University of Health and Science, Houston, USA; 3 Medical School, Dominican University, Chicago, USA; 4 Medical School, St. George's University School of Medicine, St. George, USA; 5 Internal Medicine, Montefiore Medical Center, Wakefield Campus, Bronx, USA; 6 Interventional Cardiology, Community First Medical Center, Chicago, USA

**Keywords:** left main coronary artery (lmca), left main coronary disease, left main occlusion in women, left main occlusion with cardiogenic shock, lmca in women, personalized medicine (pm)

## Abstract

Occlusion of the left main coronary artery (LMCA) is a highly lethal cause of myocardial infarction (MI), often referred to as the “widowmaker” due to its rapid progression and high mortality rate. Prompt diagnosis and intervention are crucial, although frequently delayed, particularly in females who tend to present with atypical symptoms. We report the case of a 57-year-old woman who arrived at the emergency department with severe epigastric pain and distress that progressed into altered mental status, initially prompting evaluation for gastrointestinal pathology. Her condition rapidly progressed to cardiogenic shock, with persistent hypotension, tachycardia, and new-onset atrial fibrillation with rapid ventricular response, despite no prior history of arrhythmia. Initial laboratory testing revealed elevated cardiac biomarkers and metabolic acidosis, and electrocardiogram (ECG) findings concerning for ST-elevation myocardial infarction (STEMI). Emergent coronary angiography revealed a 99% thrombotic occlusion of the LMCA. The patient underwent successful placement of a drug-eluting stent and initiation of intra-aortic balloon pump (IABP) support, leading to hemodynamic stabilization and transfer to a tertiary care center for advanced cardiac management. Despite these interventions, the patient expired due to complications of her acute MI. This case highlights the diagnostic and clinical challenges associated with LMCA occlusion, particularly in females whose symptoms often deviate from classical presentations. It emphasizes the importance of maintaining a high index of suspicion for acute coronary syndrome (ACS) even in the absence of chest pain, especially when patients exhibit signs of hemodynamic instability or arrhythmia. The presence of atrial fibrillation further complicated both the clinical picture and management strategy. Timely recognition, rapid activation of a STEMI protocol, and emergent percutaneous coronary intervention (PCI) were instrumental in stabilizing this patient for transport. More broadly, this case underscores the ongoing need for sex-specific approaches in cardiovascular medicine and reinforces the importance of early, personalized intervention strategies in high-risk presentations.

## Introduction

Left main coronary artery (LMCA) occlusion is one of the deadliest and most urgent causes of acute myocardial infarction (MI), giving it the term “widowmaker." This large coronary artery and its branches are the main source of myocardial perfusion, representing roughly 64% of myocardial blood supply [1]. While confirmed LMCA occlusions often warrant immediate clinical intervention due to their prognostic gravity, these presentations may develop subtly or be misinterpreted, particularly in females. Classic symptoms such as chest, jaw, and shoulder pain are often reported as the primary symptom in males. By contrast, females still experience classical symptoms, but more often than males present with atypical symptoms such as nausea, vomiting, dizziness, and fear of death [2]. In addition, female patients are more likely to experience prodromal symptoms for prolonged periods before acute MI presentation [2]. These subtle symptoms may lead to diagnostic ambiguity and a delay in treatment of ST-elevated myocardial infarctions (STEMI). Despite public awareness efforts and gender‑focused research, cardiovascular disease in females remains underdiagnosed, undertreated, and misunderstood, particularly in high‑risk contexts like LMCA occlusion [3]. 

Imaging technology has greatly improved in coronary artery disease; however, there is room for future techniques that assess intraluminal plaque characteristics rather than just luminal stenosis, particularly in the LMCA, where early rupture carries catastrophic consequences [4]. Further advancement techniques include, but are not limited to, optical coherence tomography (OCT) and intravascular ultrasound (IVUS). OCT is a non-invasive, highly advanced imaging technique that uses light to capture high-resolution images, while IVUS is a more invasive technique that is placed within the coronary blood vessels and is able to capture an image of the inside walls of the artery [5].

In these cases, surgical revascularization techniques are considered more beneficial than medical management alone, although much debate still exists on the preference between percutaneous interventions versus bypass grafting [6]. While there is no unified algorithm for revascularization decision making in left main artery disease, a personalized approach may be achieved through various scoring systems such as the European System for Cardiac Operative Risk Evaluation (EuroSCORE II), the Society of Thoracic Surgeons (STS) score, the Canadian Cardiovascular Society (CCS), and the anatomical complexity SYNTAX score [6,7].

We demonstrate a case of a middle-aged female presenting with non-radiating epigastric pain and signs of cardiogenic shock who was found to have atrial fibrillation and a thrombosed LMCA, necessitating urgent stent placement. This case underscores critical issues in contemporary cardiology: the limitations of current diagnostic approaches to identify high-risk coronary lesions in patients with atypical presentations, the ongoing underrecognition of cardiovascular disease in females, and the urgent need for personalized preventive strategies.

## Case presentation

A 57-year-old Polish female presented to the emergency department (ED) in significant distress with intermittent abdominal pain for several weeks, which increased significantly in severity over the last 30 minutes. The patient characterized this sensation as if her “stomach (were) coming out (of her) throat.” She described acute, sudden-onset, severe epigastric pain radiating diffusely across the abdomen, which began the prior night. She rated the pain 10/10. Although she had experienced similar episodes before, they were less intense and did not warrant emergent attention. Three days prior, she visited an urgent care center where she was advised to follow up with her primary care physician after receiving acetaminophen without symptom relief. 

Upon initial assessment, the patient denied chest pain, shortness of breath, dysuria, or fever. However, during history-taking and physical examination, she began vomiting. The patient appeared ill and in significant distress; thus, she refused to ambulate due to the severity of pain and required a wheelchair. The patient’s past medical history included gastroesophageal reflux disease, generalized anxiety disorder, and a history of tobacco use. Medications include clonazepam 0.5 mg orally twice per day as needed, diclofenac 75 mg orally twice per day, and pantoprazole 20 mg orally daily. She denied alcohol use, recreational drug use, and has no known history of deep vein thrombosis, pulmonary embolism, or prior abdominal surgeries. 

Initial vital signs revealed a heart rate of 120 beats per minute and blood pressure of 97/59 mmHg. Physical exam showed diffuse abdominal tenderness, particularly in the epigastric region, without costovertebral angle tenderness bilaterally. The patient was alert and oriented to person, place, and time. 

Approximately one hour later, the patient began experiencing chest pain and persistent nausea with further episodes of emesis. Auscultation at this time revealed normal heart sounds without murmurs, gallops, or rubs. She remained tachycardic at 140 beats per minute. Pulmonary exam showed normal effort and breath sounds with no respiratory distress. Bowel sounds were present and normal; the abdomen was soft and without guarding, distention, or palpable masses. Extremities showed no edema. Her skin was warm, pallor was noted, and capillary refill time was approximately two seconds. Although she was not diaphoretic, the overall clinical picture was concerning for cardiogenic shock. 

Repeat vitals showed a blood pressure of 97/73 mmHg, heart rate of 137 beats per minute, 20 breaths per minute, oxygen saturation of 93%, and an afebrile temperature. On repeat physical examination, the patient was in mild distress with no jugular venous distention. Bilateral ronchi were heard on auscultation. Cardiac exam this time noted an irregular rhythm. The abdomen remained soft and nontender; extremities showed no edema. Initial laboratory results (Table 1) revealed elevated cardiac enzymes, high AST, ALT, and alkaline phosphatase. 

**Table 1:** Pertinent laboratory values

	Patient’s values	Reference range
Total protein (g/dL)	6.7	6.0–8.3
Glucose (mg/dL)	256	70–99
Blood urea nitrogen (mg/dL)	23	7–20
Creatine (mg/dL)	0.93	0.6–1.3
Sodium (mmol/L)	137	135–145
Potassium (mmol/L)	3.7	3.5–5.1
Chloride (mmol/L)	105	98–107
Carbon dioxide (mmol/L)	22	22–29
Anion gap (mmol/L)	10	7–15
Blood urea nitrogen/creatine ratio	24	10-20
Calcium (mg/dL)	9.7	8.5–10.2
Albumin (g/dL)	4.3	3.5–5.0
Aspartate aminotransferase (U/L)	427	10–40
Alanine aminotransferase (U/L)	374	7–56
Alkaline phosphatase (U/L)	116	44–147
Total bilirubin (mg/dL)	0.4	0.1–1.2
Glomerular filtration rate (mL/min/1.73m²)	>60	≥60
Lipase (U/L)	37	0–160
Magnesium (mg/dL)	2.0	1.7–2.2
White blood cell (10³/µL)	9.0	4.0–11.0 ×10³
Red blood cell (10⁶/µL)	4.28	4.2–5.4 ×10⁶
Hemoglobin (g/dL)	14.1	13.5–17.5
Hematocrit (%)	40.7	38.8–50.0
Mean corpuscular volume (fL)	95	80–100
Mean corpuscular hemoglobin (pg)	33	27–34
Mean corpuscular hemoglobin concentration (g/dL)	34.7	32–36
Red cell distribution width (%)	14	11.5–14.5
Platelets (10³/µL)	278	150–450
Mean platelet volume (fL)	7.8	7.5–11.5
Troponin high sensitivity (ng/mL)	256	<0.04

**Figure 1:**
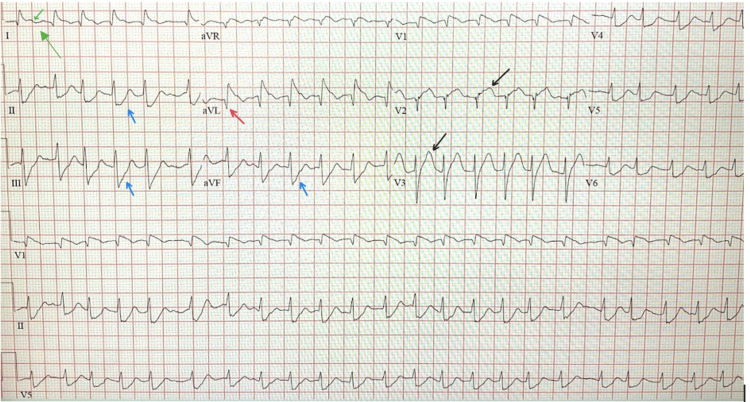
Initial EKG illustrating atrial fibrillation with rapid ventricular response with a rate of 148 (indicated by the green arrow), marked ST elevations in aVL, V1-V2 (indicated by the black arrows), and ST depression in II, III, aVF leads (indicated by the blue arrows), thus highlighting subendocardial injury with septal and lateral injury (indicated by the red arrow).

At this time, differential diagnoses included acute coronary syndrome (ACS), ruptured peptic ulcer, cholelithiasis, pancreatitis, and aortic dissection. An initial electrocardiogram (ECG), which is shown in Figure 1, showed a possible acute ST-elevation myocardial infarction (STEMI) and atrial fibrillation with rapid ventricular response, with no prior EKGs available for comparison. The patient had no prior history of atrial fibrillation or any other arrhythmias. 

**Figure 2:**
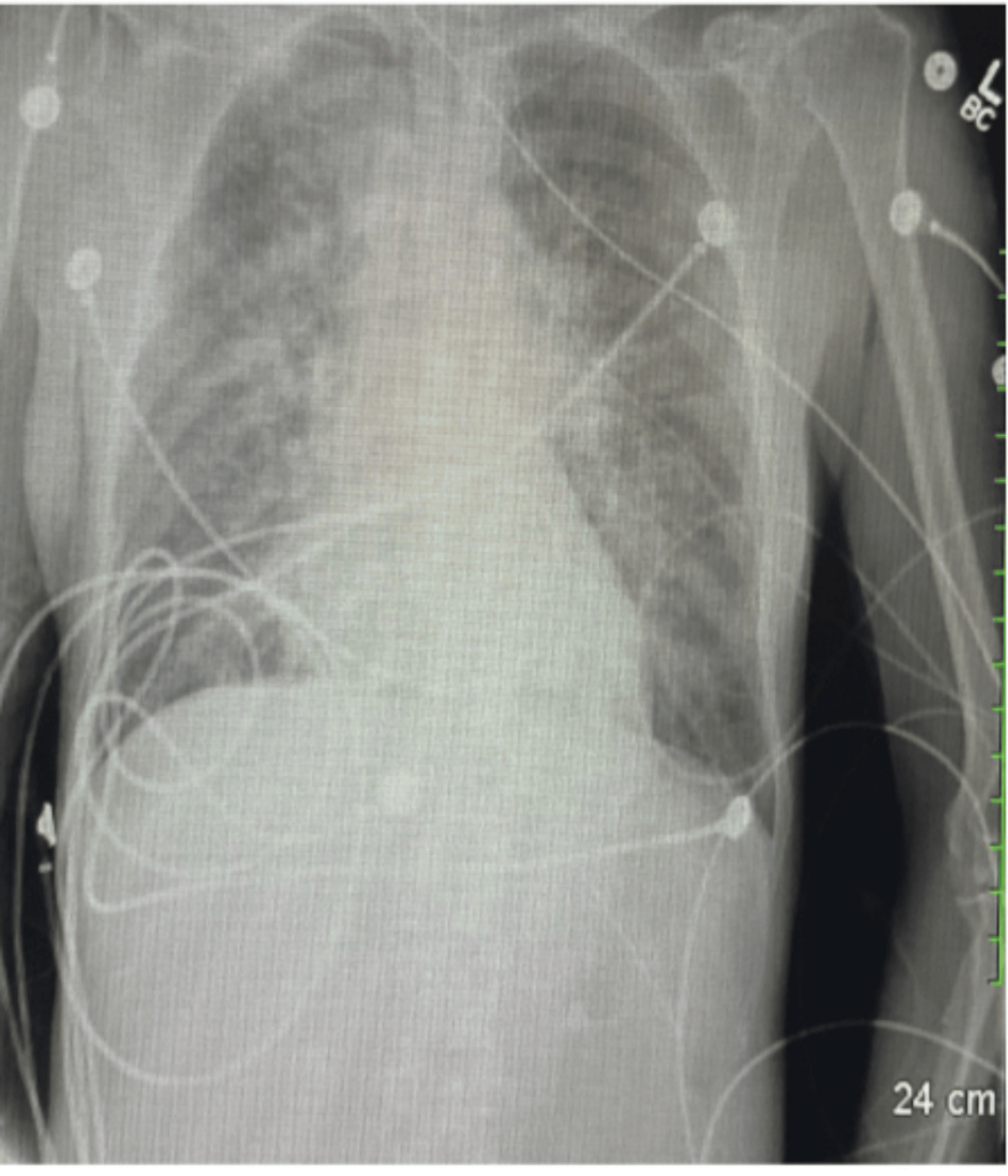
Repeat chest X-ray portable AP. Endotracheal tube is in satisfactory position. Bilateral interstitial opacities representing congestion. There is no evidence of pneumothorax or pleural effusion. Cardiomegaly was noted. Osseous structures appear to be intact, although partially visualized. AP: anterior posterior

**Figure 3:**
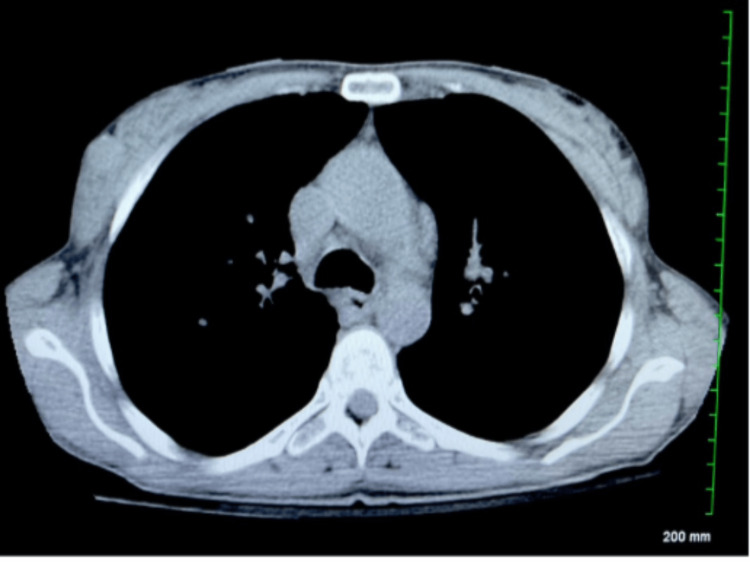
Initial chest CT angiogram without contrast CT: computed tomography

Cardiology was immediately consulted, a STEMI code was activated, and the patient was intubated. Post-intubation chest X-ray (Figure 2) for endotracheal tube placement revealed cardiomegaly and bilateral interstitial opacities. At this time, aspirin was administered, and the patient was placed on monitoring pads. Her blood pressure remained low with systolic values in the 90-100 range. A chest and abdominal computed tomography (CT) scan (chest CT shown in Figure 3) was ordered en route to the cardiac catheterization lab to rule out aortic dissection and intra-abdominal pathology; these investigations were negative for the aforementioned pathologies.

**Figure 4:**
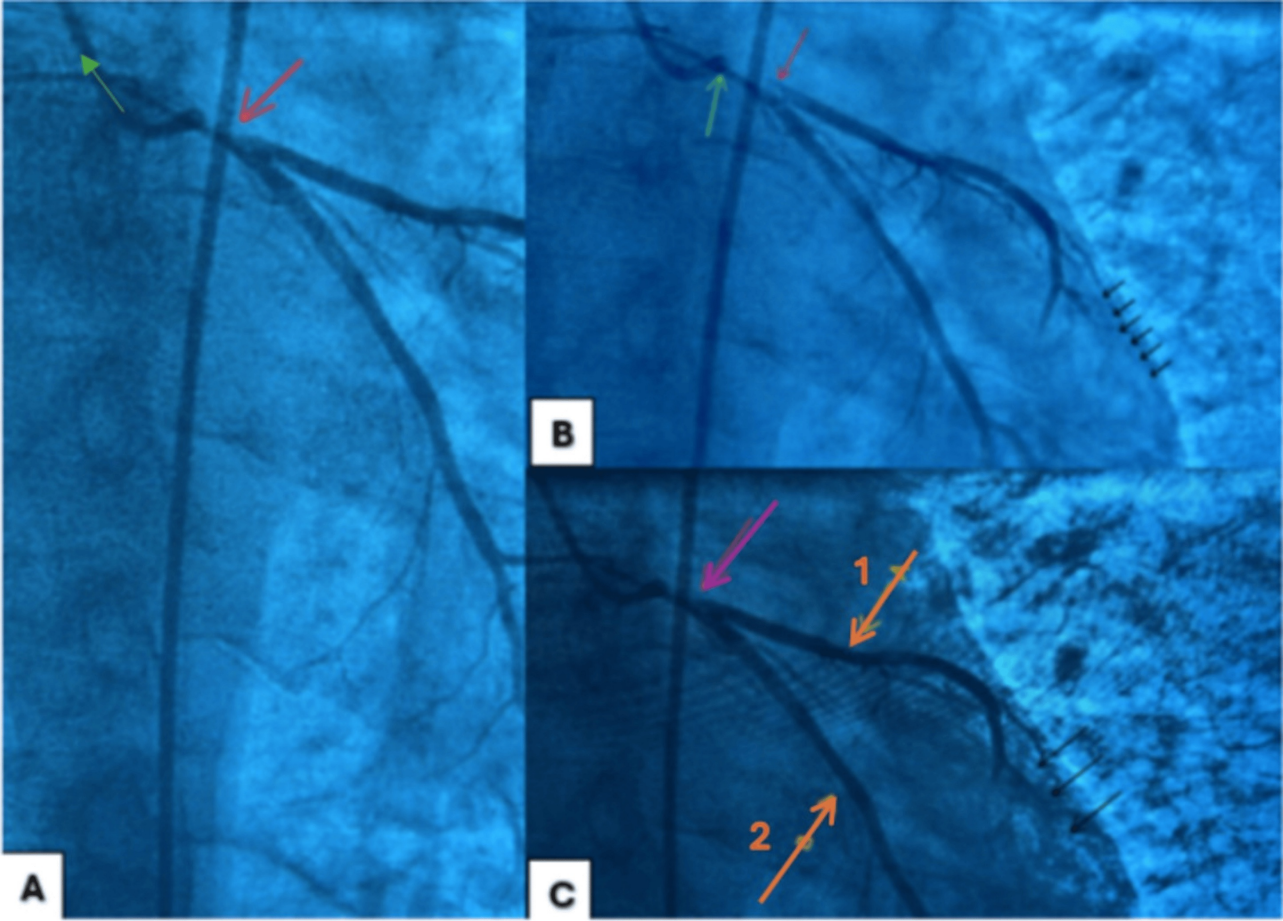
Cardiac catheterization lab: percutaneous intervention pre-stent placement. 4A: Red arrow indicating lesion on the left main coronary artery resulting in 99% stenosis of the artery. 4B: Left anterior descending artery (LAD) is not well visualized, illustrating mild diffuse disease. Red arrow: lesion on the left main coronary artery resulting in 99% stenosis of the artery. Green arrow: Catheter contrast delivery system. 4C: The right coronary artery is not well visualized (However, this patient is right dominant). Purple arrow: lesion of the left main coronary artery resulting in 99% stenosis of the artery. Orange arrow (1): LAD shows hypoperfusion of the anterior wall of the myocardium, once again highlighting diffuse disease. Orange arrow (2): LCX artery shows mild diffuse disease. LAD: left anterior descending, LCX: left circumflex

Emergent cardiac catheterization revealed complete thrombosis of the left main coronary artery, with stenosis of 99%, shown in Figure 4. Due to the critical nature of this finding, the decision was made to insert an intra-aortic balloon pump (IABP) and proceed with successful stenting. Following administration of 9000 units of heparin, a guidewire was advanced, and the left main coronary artery was stented using a 3.5 x 15 mm drug-eluting stent.

**Figure 5:**
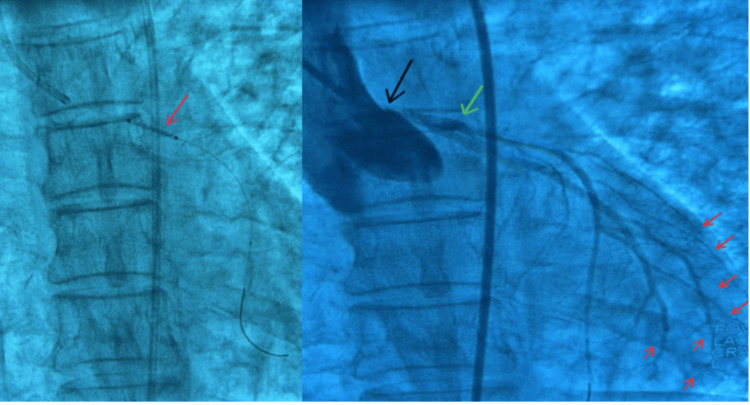
Cardiac catheterization lab images. A: The red arrow demonstrates PCI stent placement before coronary blood flow was restored. B: Percutaneous intervention post-stent placement showing flow restoration to the left system (as indicated with the red arrows). This can be compared to Figure 4 in which the LAD showed distinct hypoperfusion with diminished flow. The black arrow indicates contrast. The green arrows demonstrate stent patency.

Coronary flow was successfully restored after PCI, as shown in Figure 5. Due to the absence of intravascular ultrasound, further manipulation was deferred to minimize procedural risk. 

The patient was transferred to the intensive care unit, and due to her unstable condition, she was mechanically ventilated with continuous cardiac monitoring and frequent laboratory investigations due to the acuity and complexity of her condition. Hemodynamic status improved post-stenting. Transfer was arranged to a tertiary shock center for cardiothoracic surgery evaluation at 02:05 AM, and the patient arrived at the receiving hospital at 03:09 AM. The diagnostic delay due to atypical presentation ultimately led to rapid hemodynamic deterioration at the receiving hospital despite appropriate coronary intervention at the transferring hospital. The patient passed away the same day of transfer at the receiving hospital.

## Discussion

This case highlights an atypical presentation of MI manifesting primarily as epigastric pain and progressive altered mental status, a combination that can obscure the underlying cardiac etiology. The patient’s initial complaints of abdominal discomfort, nausea, and vomiting are more commonly associated with gastrointestinal pathology, leading to diagnostic ambiguity. Such presentations are disproportionately seen in females and can result in delayed recognition and treatment of ACS [8].

The absence of classical chest pain in this case reflects how MI presentations differ symptomatically based on biological sex. While chest pain remains the most frequent symptom in both sexes, females are significantly more likely to report atypical symptoms such as epigastric pain, fatigue, dizziness, and gastrointestinal upset [9,10]. These differences often contribute to misdiagnosis or delayed diagnosis in females, which is associated with higher morbidity and mortality, as 52% of women with ischemic heart disease will die from sudden cardiac death before reaching the hospital, as opposed to 42% in men [11].

In this case, a clinical picture that included persistent hypotension, tachycardia, atrial fibrillation with rapid ventricular response, and ECG changes prompted appropriate cardiac evaluation. ECG findings in LMCA occlusion can be nonspecific or even deceptively normal early in the disease, particularly when baseline comparisons are unavailable [12]. The development of atrial fibrillation added further diagnostic complexity, as it is both a consequence of myocardial ischemia and an independent contributor to hemodynamic instability. Atrial fibrillation can develop in about 6-15% of patients with STEMI, which can lead to further in-hospital complications throughout the treatment course [13]. New-onset atrial fibrillation is associated with worse left ventricular function, increased heart failure rates, cardiogenic shock, mechanical complications, major bleeding, stroke, and sudden cardiac death [13]. 

Coronary angiography confirmed complete thrombosis of the LMCA, a finding that carries one of the highest mortality rates among all coronary lesions [14]. The LMCA supplies most of the left ventricular myocardium, and its occlusion typically results in rapid deterioration and cardiogenic shock without emergent revascularization [15]. In-hospital mortality for patients with complete LMCA occlusion is 58% and even higher in patients who present with STEMI at 61% [16]. Urgent percutaneous coronary intervention (PCI) with IABP support facilitated hemodynamic stabilization in this patient. Mechanical circulatory support (MCS) in LMCA occlusion is consistent with guideline-directed therapy in cases of profound left ventricular failure and cardiogenic shock [17]. Although PCI with IABP while on MCS carries a higher risk of in-hospital mortality at 67%, this number may be confounded with a higher incidence of cardiogenic shock in patients using MCS [16].

IABP has been a staple treatment in cardiology for over 30 years, allowing for rapid reperfusion of the damaged myocardium and increased cardiac output. However, IABP use is decreasing due to the findings of the SHOCK-2 trial, which showed that IABP treatment was not associated with any decrease in mortality, even in patients with cardiogenic shock and early revascularization [18]. IABP was considered in the patient due to the high degree of LMCA occlusion and the diagnostic delay as a consequence of the atypical presentation.

Women who suffer from ACS and eventually MI face distinct risk profiles and treatment challenges, which warrant a more personalized approach to secondary prevention. Although evidence-based therapies such as antiplatelet agents, high-intensity statins, beta-blockers, and ACE inhibitors are proven to be effective in both sexes, females are less likely than males to receive these medications or invasive interventions despite often presenting later and with a greater comorbidity burden [19]. Tailoring therapy to individual risk factors and physiological responses is essential, especially in young women (<55 years old) who are seeing a rise in acute MI rates from 21% to 31% in a 10-year period [19]. One example could be a more aggressive blood pressure regimen since females tend to reach a target blood pressure goal (45%) than their male counterparts (39%), despite females not receiving blood pressure therapy as often as males [20]. 

Personalized cardiovascular imaging is increasingly being used not only for diagnosis but also for guiding risk reduction and preventive strategies in ACS. Future guidelines could be adapted to include intravascular modalities such as optical coherence tomography (OCT) and intravascular ultrasound (IVUS), which can identify high-risk plaque morphologies such as thin-cap fibroatheromas, plaque rupture, and erosion that may benefit from intensified antiplatelet therapy, lipid-lowering agents, or closer follow-up [21]. While these modalities are more invasive and expensive, through combining these imaging insights with individualized pharmacologic and lifestyle interventions, physicians can more effectively prevent recurrent ischemic events and tailor long-term management to each patient’s unique risk profile [22]. 

A structured cardiac rehabilitation program that is tailored to both the physician's and patient's goals is crucial in decreasing any MI recurrence or cardiac-related mortality. Programs should include exercise plans, dietary counseling, and lifestyle modification, such as alcohol and smoking cessation. Both women and men have seen substantial benefits with cardiac rehabilitation programs tailored to their needs compared to when using a more guideline-oriented approach, with an increased adherence to the program in both males and females [20]. Patients in an exercise-based cardiac rehabilitation program saw a reduction in cardiac mortality when compared to a no-exercise control (relative risk ratio of 0.74) [22].

The patient's eventual transfer to a tertiary care center and eventual passing emphasize the importance of coordinated care pathways in high-risk ACS. Early recognition, rapid activation of STEMI protocols, prompt coronary catheterization, and appropriate escalation of care are instrumental in achieving a favorable outcome. This case serves as a reminder that clinicians must maintain a high index of suspicion for acute coronary syndrome in patients presenting with nonspecific or atypical complaints, particularly in populations at risk for underrecognized cardiovascular disease.

## Conclusions

This case emphasizes the diagnostic challenges that arise from atypical presentations, particularly in female patients, where classical symptoms such as chest pain may be absent and instead manifest as gastrointestinal discomfort, nausea, or altered mental status. This case stands to emphasize the importance of awareness of these atypical symptoms of MI. Furthermore, the patient's rapid clinical deterioration and ultimate diagnosis of a 99% thrombotic LMCA occlusion highlight the necessity for healthcare workers to maintain a high index of suspicion for cardiac pathology in females presenting with nonspecific, yet severe systemic symptoms. Consequently, the prompt activation of the STEMI protocol, emergent PCI, and initiation of MCS were instrumental in achieving hemodynamic stabilization and facilitating tertiary care transfer. 

The patient’s persistent hypotension, tachycardia, and new-onset atrial fibrillation with nonspecific ECG changes obscured the early recognition of left main coronary artery occlusion, underscoring the need for heightened clinical suspicion given its association with poor in-hospital outcomes. While IABP utilization with PCI has decreased in recent years, it was required in this case due to the high degree of coronary occlusion and delayed intervention. Women with ACS and MI face unique risk factors and underutilization of evidence-based therapies, making personalized approaches, including targeted pharmacologic regimens, advanced cardiovascular imaging, and tailored cardiac rehabilitation, essential for improving prevention and long-term outcomes. This case contributes to the growing literature calling for enhanced diagnostic vigilance, tailored treatment pathways, and equity in cardiovascular care delivery, particularly in high-mortality entities such as LMCA occlusion. 
